# Perception of strength, attractiveness and aggressiveness of Maasai male faces calibrated to handgrip strength: Evidence from a European sample

**DOI:** 10.1002/ajhb.23869

**Published:** 2023-01-24

**Authors:** Sonja Windhager, Theresa Ottendorfer, Audax Mabulla, Marina Butovskaya, Bernhard Fink, Katrin Schaefer

**Affiliations:** ^1^ Department of Evolutionary Anthropology University of Vienna Vienna Austria; ^2^ Human Evolution and Archaeological Sciences (HEAS) University of Vienna Vienna Austria; ^3^ Department of Archaeology University of Dar es Salaam Dar es Salaam Tanzania; ^4^ Institute of Ethnology and Anthropology Russian Academy of Sciences Moscow Russian Federation; ^5^ Social Anthropology Research and Education Center Russian State University for Humanities Moscow Russian Federation; ^6^ Faculty of Humanities National Research University Higher School of Economics Moscow Russian Federation; ^7^ Biosocial Science Information Biedermannsdorf Austria

## Abstract

**Objectives:**

Previous research showed that male and female members of the Maasai from Northern Tanzania judge images of facial morphs calibrated to greater handgrip strength (HGS) higher on strength and attractiveness, but lower on aggressiveness than those calibrated to lower HGS. The accurate assessment of male physical strength from facial information may be adaptive as suggested by the evidence on health and fitness‐related benefits linked to high muscular strength.

**Methods:**

This study extends previous work by obtaining European female (*n* = 220) and male (*n* = 51) assessments of HGS‐calibrated Maasai male faces. Participants rated five facial morphs for strength, attractiveness, and aggressiveness on computer screens.

**Results:**

Perceived physical strength increased with morphs calibrated to higher HGS. The lowest and highest HGS morphs were judged lower in attractiveness than the others, and rated aggressiveness decreased in morphs calibrated to higher HGS.

**Conclusions:**

Given the high similarity between the current study findings and those previously reported from intra‐population assessments of Maasai faces calibrated to HGS, we suggest that strength and aggressiveness perceptions of facial features associated with male physical strength may be universal. Attractiveness assessments of strength‐related information in the faces of (very) strong men were less consistent across populations, possibly attributable to cultural and ecological contexts.

## 1 INTRODUCTION

Handgrip strength (HGS) is a proxy for overall muscular strength (Wind et al., 2010) and function that correlates positively with physical fitness and negatively with morbidity and mortality in both sexes (Cooper et al., 2010). However, HGS is highly sexually dimorphic with men—on average—being physically stronger than women. This dimorphism maps onto heritability estimates for HGS (male > female) and the androgenic influences in the development of HGS (Isen et al., 2014).

Increased physical strength may have favored ancestral males in intrasexual competition (Sell et al., 2009) and hunting (Apicella, 2014). Previous research has shown that in men, HGS is associated with body morphology, sexual behavior, and measures of intrasexual competition (Gallup et al., 2007). Men and women can accurately assess physical strength from images of male faces and bodies (Sell et al., 2009) and consider the faces of physically strong men as attractive (Butovskaya et al., 2022; Fink et al., 2007).

Most of the evidence for perceptual correlates of HGS has been derived from Western samples (see for review, Gallup & Fink, 2018). Recent research, however, documents HGS relationships with face shape also in male members of a small‐scale society, that is, the Maasai of Northern Tanzania, suggesting that the facial correlates of physical strength could be universal (Butovskaya et al., 2018). In a follow‐up study (Butovskaya et al., 2022), Maasai women and men judged Maasai male facial morphs calibrated to greater HGS higher on strength and attractiveness, but lower on aggressiveness than those calibrated to average or lesser HGS. This suggests that Maasai are sensitive to facial cues of strength and use them in social assessments.

Here, we extend these findings by providing cross‐cultural evidence from European raters. Thus, the current study aimed to examine strength, attractiveness, and aggressiveness assessments of Maasai male facial morphs calibrated to HGS (as used in Butovskaya et al., 2022) in a sample from an industrialized society.

## 2 MATERIAL AND METHODS

### 2.1 Participants

Female (*n* = 220) and male (*n* = 51) assessors were individually recruited in Austria in May/June 2018 and 2020. The age of female participants ranged from 18 to 36 years (*Md* = 23, *SIR* = 2.5), 87% reported to be students, 46% had at least one parent from Austria, further 52% had both parents from Europe. The age of male participants ranged from 20 to 35 years (*Md* = 27, *SIR* = 2.5), 55% reported to be students, 71% had at least one Austrian parent, 22% had both parents from Europe. Informed consent was obtained from all participants.

### 2.2 Stimuli

A geometric morphometrics approach was used for stimulus creation (Windhager et al., 2018). The calibration sample comprised facial images and HGS measures (in kgf) of 54 young‐adult Maasai men (20–29 years) from the Ngorongoro area in Northern Tanzania. The regression of these facial shapes on HGS (Butovskaya et al., 2018) provided target configurations for image unwarping and averaging resulting in five calibrated morphs: The sample average and two toward the extrema of the empirical shape variation along the regression vector (here ±5 SD of HGS from that average, as rated intra‐culturally in Butovskaya et al., 2022) together with two intermediate shapes (±2 SD of HGS).

### 2.3 Rating study

The stimuli were presented on screen using the SoSci Survey platform (https://www.soscisurvey.de). Sliders ranging from low to high on the attribute (0–100, values concealed) were presented next to each facial morph. Participants were asked to rate them for physical strength, attractiveness, and aggressiveness, respectively. The order of presentation of the five morphs was randomized across participants.

### 2.4 Statistical analysis

Friedman tests, together with pairwise Wilcoxon tests, were performed to examine differences in assessments (separately for each attribute) between the five facial morphs. The *p*‐values are reported as two‐tailed and uncorrected. Boxplots show median and interquartile ranges as well as whiskers without outliers (circles). The analysis was performed in IBM SPSS 27.

## 3 RESULTS

**FIGURE 1:**
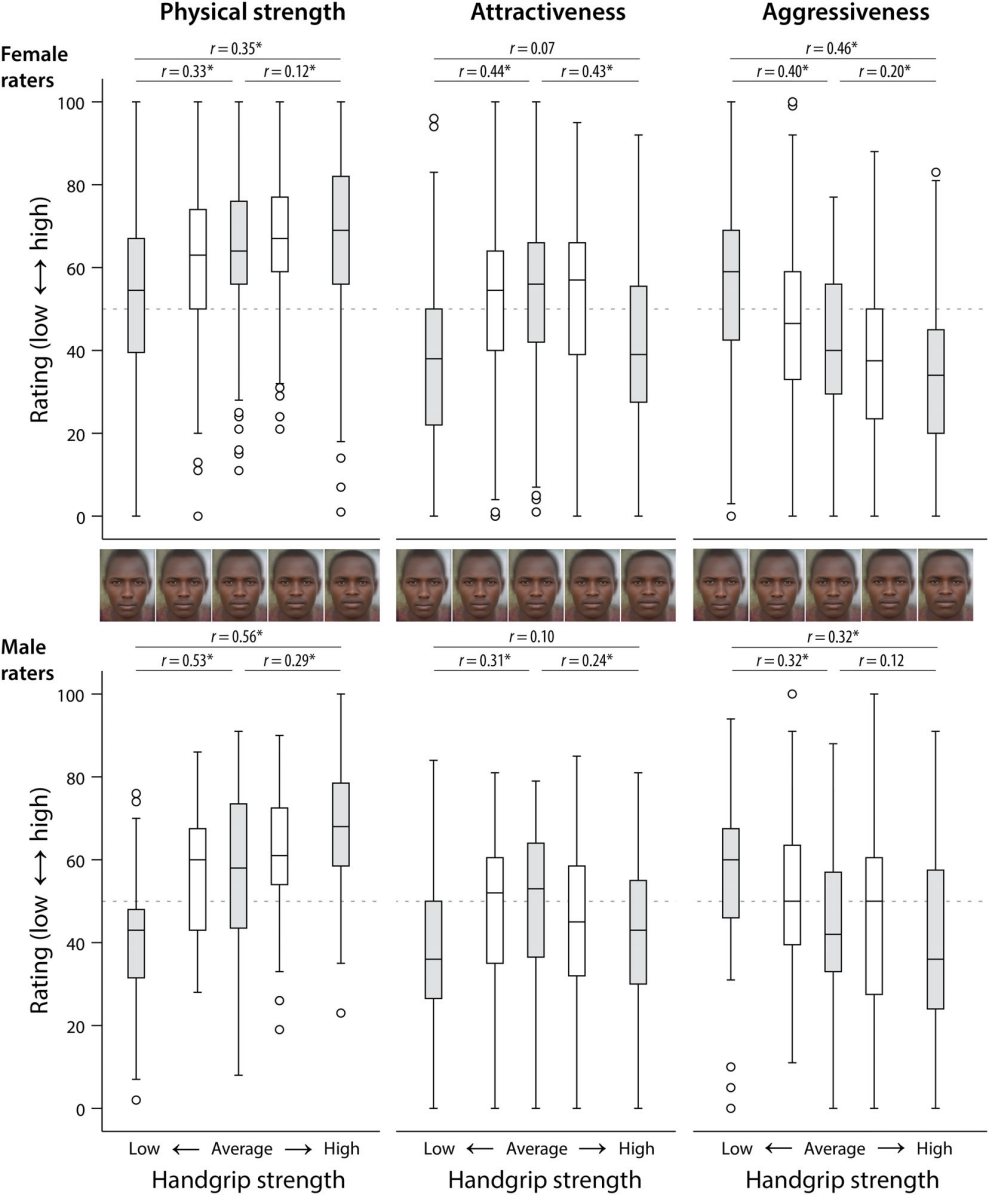
Assessments of Maasai male facial morphs calibrated to handgrip strength by female and male members of a European sample. Averages and extrema (±5 SD) are in gray. Effect sizes (*r*s) are provided for pairwise comparisons between extrema (longer horizontal brackets), and between average and extrema (shorter brackets).

There were distinct response patterns for the three attributes across facial morphs: perceived physical strength increased in morphs calibrated to higher HGS, perceived attractiveness showed a cap‐shaped pattern with the lowest and highest HGS morphs judged less positively than others, and perceived aggressiveness decreased in morphs calibrated to higher HGS (Figure 1). Friedman tests indicated differences between the morphs for the three attributes: physical strength (female *χ* ^2^ = 87.4, *p* = 4.6E−18; male *χ* ^2^ = 54.5, *p* = 4.1E−11), attractiveness (female χ^2^ = 161.0, *p* = 8.6E−34; male χ^2^ = 22.7, *p* = 1.5E−4), and aggressiveness (female χ^2^ = 170.0, *p* = 1.1E−35; male χ^2^ = 22.2, *p* = 1.9E−4). Table A1 provides the statistics for all pairwise comparisons.

Out of the 15 possible gender comparisons, 14 were nonsignificant after Bonferroni adjustment (Table A2). Women rated the weakest morph higher in physical strength than men did.

## 4 DISCUSSION

In a comprehensive review, Puts (2010) hypothesized that in ancestral humans the accurate assessment of strength has been crucial in social encounters, as men had to be sensitive to strength‐related features in possible rivals, and women may have developed a preference for strong men who outcompete their counterparts. At the proximate level, both HGS and facial traits share a common substrate. That is, genetic, prenatal and pubertal effects of androgens are supposed to influence the development of facial shape, male muscularity and physical strength as do individual age and developmental factors such as work and leisure activities (Butovskaya et al., 2018; Isen et al., 2014).

Windhager et al. (2011) utilized a geometric morphometric approach to disentangle the components that contribute to (female) perceptions of European male physical strength, attractiveness, dominance and masculinity. Facial features associated with high HGS in a sample of European men were a more robust face with a rounded outline, relatively wider eyebrows, and a more well‐curved jaw than in weaker men. A similar pattern of facial morphology associated with HGS was reported for Maasai (Butovskaya et al., 2018)—images which have been employed in the present study. In short, the geometric morphometric analysis suggests that facial shape associated with physical strength transcends populations. This may explain why previous studies have reported consistent relationships of physical strength with several behavioral and perceived traits (see for review, Gallup & Fink, 2018).

Facial images of physically strong European men have been reported to be judged higher for dominance, masculinity, and attractiveness (Fink et al., 2007). Recent research documents strength‐related facial perception also among the Maasai (Butovskaya et al., 2022). Maasai men and women judged facial morphs calibrated to greater HGS higher on strength and attractiveness, but lower on aggressiveness than faces calibrated to lesser HGS. The current study used the same strength‐calibrated facial morphs and found the same overall response patterns for European raters. Possible explanations for the negative strength‐aggressiveness relationship in social face perception include (i) feature‐based perceptual overgeneralization of aggressiveness from facial strength cues (see Oosterhof & Todorov, 2008), due to, for example, structural similarities between facial correlates of low physical strength and angry frowns, and (ii) a kernel of truth interpretation with aggressiveness being a facultative behavioral strategy of weaker men to compensate for physical disadvantage in resource competition (Knapen et al., 2018).

Facial attractiveness attributions increased with higher HGS in both populations, albeit very high HGS reduced attractiveness ratings among Europeans, but pushed them higher among Maasai. The Darwinian perspective predicts a fitness‐dependent relationship between physical strength and preference (attractiveness), and evidence confirms that physical strength is an important component of status and reputation, especially for young men (Gallup & Fink, 2018). Taken together, the findings of the present study suggest that (i) previous reports on the ability to perceive physical strength from facial morphology are genuine, and (ii) the assessments of strength and aggressiveness are similar across sex and populations. Attractiveness assessments of strength‐calibrated faces, however, may not show coherent relationships across populations for faces of very strong men. Cross‐cultural variability in ratings of attractiveness more than for other attributes has previously been documented (e.g., Voegeli et al., 2021) and may originate from population‐specific socio‐cultural settings (e.g., the “junior warriors” in the Maasai age‐set system) and environmental contexts, such as national health (DeBruine et al., 2010).

## AUTHOR CONTRIBUTIONS

**Sonja Windhager:** Conceptualization (equal), data curation (equal), formal analysis (lead), funding acquisition (equal), software (lead), writing—original draft (equal), writing—review and editing (equal). **Theresa Ottendorfer**: Conceptualization (supporting), investigation (equal), formal analysis (supporting). **Audax Mabulla**: Resources (equal), writing—review and editing (equal). **Marina Butovskaya**: Data curation (equal), funding acquisition (equal), investigation (equal), resources (equal), writing—review and editing (equal). **Bernhard Fink**: Conceptualization (equal), writing—original draft (equal), writing—review and editing (equal). **Katrin Schaefer**: Conceptualization (equal), funding acquisition (equal), resources (equal), software (supporting), writing—review and editing (equal).

## FUNDING INFORMATION

Marina Butovskaya conducted this work in accordance with the research plans of the Institute of Ethnology and Anthropology RAS; Austrian Science Fund, FWF P29397 and Faculty of Life Sciences, University of Vienna, Young Investigator Award (Sonja Windhager); German Science Foundation, DFG FI1450/7‐2 (Bernhard Fink).

## CONFLICT OF INTEREST

The authors declare no conflict of interest.

**TABLE A1:** Test statistics of the pairwise Wilcoxon tests for comparing the ratings of the five facial morphs by gender. The reported *p*‐values are two‐tailed and uncorrected.

		Pair of morphs (Handgrip strength)									
		Very low—Low	Very low—Average	Very low—High	Very low—Very high	Low—Average	Low—High	Low—Very high	Average—High	Average—Very high	High—Very high
Gender	Statistics^a^			Physical strength rating							
Female	*Z*	−5.626^b^	6.971^b^	−7.517^b^	−7.277^b^	−1.364^b^	−3.626^b^	−3.520^b^	−2.626^b^	−2.559^b^	1.114^b^
	*r*	0.268	0.332	0.358	0.347	0.065	0.173	0.168	0.125	0.122	0.053
	*p*	1.841 E−08	3.156 E−12	5.586 E−14	3.410 E−13	0.173	2.881 E−04	4.324 E−04	0.009	0.011	0.265
Male	*Z*	−4.577^b^	−5.325^b^	−5.198^b^	−5.638^b^	−1.072^b^	−2.050^b^	−3.244^b^	−1.752^b^	−2.935^b^	−2.473^b^
	*r*	0.453	0.527	0.515	0.558	0.106	0.203	0.321	0.173	0.291	0.245
	*p*	4.717 E−06	1.009 E−07	2.013 E−07	1.720 E−08	0.284	0.040	0.001	0.080	0.003	0.013
				Attractiveness rating							
Female	*Z*	−8.393^b^	−9.255^b^	−8.248^b^	−1.554^b^	−2.468^b^	−0.646^b^	−6.695^c^	−1.195^c^	−9.195^c^	−8.168^c^
	*r*	0.400	0.441	0.393	0.074	0.118	0.031	0.319	0.057	0.438	0.389
	*p*	4.732 E−17	2.135 E−20	1.608 E−16	0.120	0.014	0.518	2.154 E−11	0.232	3.741 E−20	3.138 E−16
Male	*Z*	−3.612^b^	−3.133^b^	−1.985^b^	−0.966^b^	−0.553^b^	−1.276^c^	−1.893^c^	−1.687^c^	−2.391^c^	−2.042^c^
	*r*	0.358	0.310	0.197	0.096	0.055	0.126	0.187	0.167	0.237	0.202
	*p*	3.039 E−04	0.002	0.047	0.334	0.580	0.202	0.058	0.092	0.017	0.041
				Aggressiveness rating							
Female	*Z*	−5.976^c^	−8.469^c^	−9.296^c^	−9.635^c^	−2.742^c^	−5.172^c^	−6.189^c^	−2.757^c^	−4.138^c^	−2.170^c^
	*r*	0.285	0.404	0.443	0.459	0.131	0.247	0.295	0.131	0.197	0.103
	*p*	2.286 E−09	2.482 E−17	1.455 E−20	5.669 E−22	0.006	2.316 E−07	6.054 E−10	0.006	3.499 E−05	0.030
Male	*Z*	−1.927^c^	−3.228^c^	−2.179^c^	−3.225^c^	−1.771^c^	−1.231^c^	−2.493^c^	−0.528^b^	−1.159^c^	−1.368^c^
	*r*	0.191	0.320	0.216	0.319	0.175	0.122	0.247	0.052	0.115	0.135
	*p*	0.054	0.001	0.029	0.001	0.077	0.218	0.013	0.598	0.246	0.171

^a^ Wilcoxon signed ranks test.

^b^ Based on negative ranks.

^c^ Based on positive ranks.

**TABLE A2:** Comparison of female and male ratings per trait and handgrip strength morph.

Morph	Mean rank		Rank sum	Mean rank difference	*U*	*p* uncorr.	*p* corr. trait	*p* corr. overall		
	Male	Female							*z*	*r*
	Physical strength rating									
Very low	96.1	145.2	31953.5	49.1	3576.5	5.50 E‐05	2.75 E‐04	**0.001**	−4.033	−0.245
Low	114.3	141.0	31025.0	26.7	4505.0	0.028	0.142	0.426	−2.192	−0.133
Average	116.4	140.6	30921.5	24.2	4608.5	0.047	0.235	0.705	−1.986	−0.121
High	114.1	141.1	31038.0	27.0	4492.0	0.027	0.133	0.399	−2.218	−0.135
Very high	136.9	135.8	29875.5	−1.1	5565.5	0.930	1.000	1.000	−0.088	−0.005
	Attractiveness rating									
Very low	138.5	135.4	29792.5	−3.1	5482.5	0.800	1.000	1.000	−0.253	−0.015
Low	124.1	138.8	30525.5	14.6	5004.5	0.230	1.000	1.000	−1.201	−0.073
Average	123.3	138.9	30567.0	15.6	4963.0	0.199	0.997	1.000	−1.283	−0.078
High	110.4	141.9	31227.0	31.6	4303.0	0.010	0.048	0.143	−2.592	−0.157
Very high	142.7	134.4	29577.5	−8.3	5267.5	0.497	1.000	1.000	−0.679	−0.041
	Aggressiveness rating									
Very low	139.2	135.3	29758.0	−3.9	5448.0	0.748	1.000	1.000	−0.321	−0.020
Low	155.7	131.4	28916.0	−24.2	4606.0	0.046	0.232	0.697	−1.991	−0.121
Average	145.3	133.8	29443.5	−11.5	5133.5	0.345	1.000	1.000	−0.945	−0.057
High	162.2	129.9	28582.0	−32.3	4272.0	0.008	0.040	0.119	−2.654	−0.161
Very high	152.3	132.2	29089.0	−20.1	4779.0	0.099	0.497	1.000	−1.648	−0.100

*Note*: The table gives the mean rank for the female (*n* = 220) and the male (*n* = 51) raters together with their difference (female mean rank minus male mean rank), followed by the test statistics for the Mann–Whitney *U*‐tests. Effect sizes and their verbal interepretation follow Field (2009; Discovering statistics using SPSS, 3rd ed.). *P*‐values are two‐tailed and uncorrected (*p* uncorr.) as well as Bonferroni‐corrected for five tests per trait (*p* corr. trait), and for the overall number of 15 tests (*p* corr. overall). In the latter condition, there is only one significant gender difference in that female raters assigned higher strength to the weakest morph than the male raters. The effect size (*r*) is small. The second largest effect size is only −0.161. The box plots (Figure 1 of the article) confirm that the overall response pattern is highly similar for each trait.

## Data Availability

All relevant data are available from the public repository Phaidra (https://phaidra.univie.ac.at/o:1623082).

## References

[ajhb23869-bib-0001] Apicella, C. L. (2014). Upper‐body strength predicts hunting reputation and reproductive success in Hadza hunter–gatherers. Evolution and Human Behavior, 35(6), 508–518. 10.1016/j.evolhumbehav.2014.07.001

[ajhb23869-bib-0002] Butovskaya, M. L. , Mezentseva, A. , Mabulla, A. , Shackelford, T. K. , Schaefer, K. , Fink, B. , & Windhager, S. (2022). Facial cues to physical strength increase attractiveness but decrease aggressiveness assessments in male Maasai of northern Tanzania. Evolution and Human Behavior, 43(2), 115–121. 10.1016/j.evolhumbehav.2021.11.006

[ajhb23869-bib-0003] Butovskaya, M. L. , Windhager, S. , Karelin, D. , Mezentseva, A. , Schaefer, K. , & Fink, B. (2018). Associations of physical strength with facial shape in an African pastoralist society, the Maasai of northern Tanzania. PLoS ONE, 13(5), e0197738. 10.1371/journal.pone.0197738 29852024 PMC5978875

[ajhb23869-bib-0004] Cooper, R. , Kuh, D. , Hardy, R. , & Mortality Review Group, FALCon and HALCyon Study Teams . (2010). Objectively measured physical capability levels and mortality: Systematic review and meta‐analysis. BMJ, 341, c4467. 10.1136/bmj.c4467 20829298 PMC2938886

[ajhb23869-bib-0005] DeBruine, L. M. , Jones, B. C. , Crawford, J. R. , Welling, L. L. M. , & Little, A. C. (2010). The health of a nation predicts their mate preferences: Cross‐cultural variation in women's preferences for masculinized male faces. Proceedings of the Royal Society B: Biological Sciences, 277(1692), 2405–2410. 10.1098/rspb.2009.2184 PMC2894896 20236978

[ajhb23869-bib-0006] Fink, B. , Neave, N. , & Seydel, H. (2007). Male facial appearance signals physical strength to women. American Journal of Human Biology, 19(1), 82–87. 10.1002/ajhb.20583 17160983

[ajhb23869-bib-0007] Gallup, A. C. , & Fink, B. (2018). Handgrip strength as a Darwinian fitness indicator in men. Frontiers in Psychology, 9, 439. 10.3389/fpsyg.2018.00439 29681871 PMC5898311

[ajhb23869-bib-0008] Gallup, A. C. , White, D. D. , & Gallup, G. G. (2007). Handgrip strength predicts sexual behavior, body morphology, and aggression in male college students. Evolution and Human Behavior, 28, 423–429. 10.1016/j.evolhumbehav.2007.07.001

[ajhb23869-bib-0009] Isen, J. , McGue, M. , & Iacono, W. (2014). Genetic influences on the development of grip strength in adolescence. American Journal of Physical Anthropology, 154, 189–200. 10.1002/ajpa.22492 24936605 PMC4061497

[ajhb23869-bib-0010] Knapen, J. E. P. , Blaker, N. M. , & Van Vugt, M. (2018). The Napoleon complex: When shorter men take more. Psychological Science, 29(7), 1134–1144. 10.1177/0956797618772822 29746217 PMC6247438

[ajhb23869-bib-0011] Oosterhof, N. N. , & Todorov, A. (2008). The functional basis of face evaluation. Proceedings of the National Academy of Sciences of the United States of America, 105(32), 11087–11092. 10.1073/pnas.0805664105 18685089 PMC2516255

[ajhb23869-bib-0012] Puts, D. A. (2010). Beauty and the beast: Mechanisms of sexual selection in humans. Evolution and Human Behavior, 31(3), 157–175. 10.1016/j.evolhumbehav.2010.02.005

[ajhb23869-bib-0013] Sell, A. , Cosmides, L. , Tooby, J. , Sznycer, D. , Von Rueden, C. , & Gurven, M. (2009). Human adaptations for the visual assessment of strength and fighting ability from the body and face. Proceedings of the Royal Society B: Biological Sciences, 276(1656), 575–584. 10.1098/rspb.2008.1177 PMC2664345 18945661

[ajhb23869-bib-0014] Voegeli, R. , Schoop, R. , Prestat‐Marquis, E. , Rawlings, A. V. , Shackelford, T. K. , & Fink, B. (2021). Cross‐cultural perception of female facial appearance: A multi‐ethnic and multi‐centre study. PLOS One, 16(1), e0245998. 10.1371/journal.pone.0245998 33481957 PMC7822532

[ajhb23869-bib-0015] Wind, A. E. , Takken, T. , Helders, P. J. , & Engelbert, R. H. (2010). Is grip strength a predictor for total muscle strength in healthy children, adolescents, and young adults? European Journal of Pediatrics, 169, 281–287. 10.1007/s00431-009-1010-4 19526369

[ajhb23869-bib-0016] Windhager, S. , Bookstein, F. L. , Mueller, H. , Zunner, E. , Kirchengast, S. , & Schaefer, K. (2018). Calibrating facial morphs for use as stimuli in biological studies of social perception. Scientific Reports, 8, 6698. 10.1038/s41598-018-24911-0 29703983 PMC5923288

[ajhb23869-bib-0017] Windhager, S. , Schaefer, K. , & Fink, B. (2011). Geometric morphometrics of male facial shape in relation to physical strength and perceived attractiveness, dominance, and masculinity. American Journal of Human Biology, 23(6), 805–814. 10.1002/ajhb.21219 21957062

